# Psychosocial factors in adherence to pharmacological treatment and diabetes mellitus control in patients over 65

**DOI:** 10.1016/j.aprim.2022.102302

**Published:** 2022-04-14

**Authors:** Alfredo Lara-Morales, Ana Gandarillas-Grande, Antonio Díaz-Holgado, Pilar Serrano-Gallardo

**Affiliations:** a University of Guanajuato, Lascuráin de Retana No. 5, Guanajuato, Gto, Mexico; b Epidemiology Service, Subdirectorate of Epidemiology, General Directorate of Public Health, Madrid Primary Care Service, Madrid, Spain; c Technical Directorate of Health Information Systems, Assistant Management of Assistance Processes, Madrid Primary Care Service, Madrid, Spain; d Nursing Department, School of Medicine, Autonomous University of Madrid, IDIPHISA, INAECU, Madrid, Spain

**Keywords:** Diabetes mellitus, Type 2, Aging, Primary Health Care, Mental health, Patient compliance, Social risk, Diabetes mellitus, Tipo 2, Envejecimiento, Atención Primaria de Salud, Salud mental, Adherencia al tratamiento, Riesgo social

## Abstract

**Aim:**

To explore the influence of anxiety/depression symptoms and social risk in patients older than 65 years with type 2 diabetes mellitus (T2DM) both in non-adherence to pharmacological treatment (Non-AdhT) and in poor control of T2DM.

**Design:**

Cross-sectional study.

**Setting and participants:**

Adults over 65 years of age with T2DM treated at the Madrid Primary Care Service.

**Main measurements:**

Data collection: Electronic Health Record database. Variables: Poor control of T2DM (HBA1c) and Non-AdhT (Morisky-Green test); main clinical variables: symptoms of depression/anxiety and social risk. Global multivariate logistic regression models and disaggregated by sex were used to Non-AdhT and poor T2DM control.

**Results:**

Data were obtained on 884 subjects. Non-AdhT prevalence: 4.4%; prevalence of poor T2DM control: 37.2%. Multivariate logistic regression models for No-AdhT in men showed a higher risk if they had symptoms of anxiety/depression (OR: 3.88; 95%CI: 1.15–13.07); and in women, if they had social risk (OR: 5.61; 95%CI: 1.86–16.94). Multivariate logistic regression models for poor control of T2DM in men revealed a higher risk if they did not have AdhT (OR: 3.53; 95%CI: 1.04–12.02).

**Conclusions:**

In people over 65 years with T2DM, although Non-AdhT is low, the prevalence of poor T2DM control is high. Symptoms of depression or anxiety are a risk factor to Non-AdhT in men, while social risk has the same effect in women. Non-AdhT in men increases the risk of poor T2DM control. From a gender perspective, it is important to detect social and mental health problems in older adults with diabetes and to reinforce strategies to improve their adherence to drug treatment in these patients.

## Abstract (es)

**Objetivo:**

Explorar la influencia de los síntomas de ansiedad/depresión y del riesgo social en pacientes mayores de 65 años con diabetes mellitus tipo 2 (DM2), tanto en la no adherencia al tratamiento farmacológico (no AdhT) como en el mal control de la DM2.

**Diseño:**

Estudio descriptivo transversal.

**Emplazamiento y participantes:**

Adultos mayores de 65 años con DM2 atendidos en el Servicio de Atención Primaria de Madrid.

**Principales mediciones:**

Recogida de datos: base de datos de historia clínica electrónica. Variables: mal control de T2DM (HBA1c) y no AdhT (prueba de Morisky-Green); principales variables clínicas: síntomas de depresión/ansiedad y riesgo social. Se utilizaron modelos globales de regresión logística multivariante y desagregados por sexo para no AdhT y mal control de DM2.

**Resultados:**

Se obtuvieron datos de 884 sujetos. Prevalencia de no AdhT: 4,4%; prevalencia de mal control de DM2: 37,2%. Los modelos de regresión logística multivariante para no AdhT en hombres mostraron un mayor riesgo si tenían síntomas de ansiedad/depresión (OR: 3,88; IC del 95%: 1,15-13,07); y en mujeres, si tenían riesgo social (OR: 5,61; IC del 95%: 1,86-16,94). Los modelos de regresión logística multivariante para el control deficiente de la DM2 en los hombres revelaron un mayor riesgo si no tenían AdhT (OR: 3,53; IC del 95%: 1,04-12,02).

**Conclusiones:**

En personas mayores de 65 años con DM2, aunque la no AdhT es baja, la prevalencia de mal control de la DM2 es alta. Los síntomas de depresión o ansiedad son un factor de riesgo de no AdhT en los hombres, mientras que el riesgo social tiene el mismo efecto en las mujeres. La no AdhT en los hombres aumenta el riesgo de un control deficiente de la DM2. Desde una perspectiva de género, es importante detectar problemas de salud social y mental en adultos mayores con diabetes y reforzar estrategias para mejorar su adherencia al tratamiento farmacológico y control glucémico en estos pacientes.

## Introduction

The prevalence worldwide of diabetes mellitus (DM) in patients over 18 years of age has increased from 4.7% in 1980 to 8.5% in 2017 and is foreseen to reach 9.9% in 2045.^1^, ^2^ This disease affects people of all ages but is typically prevalent in people over 65 years of age (19.3%).^3^ In Spain, DM is a health problem that is highly prevalent in people over 65 (at the national level^4^: 21.3% from 65 to 74 years, 25% in men and 18% in women; in Madrid: 25.2% from 60 to 69 years, 32.8% in men and 18.3% in women.^5^

In the systematic review and meta-analysis conducted by Rotella and Mannuci^6^ with the aim of assessing the incidence of among people suffering from depression, while pointing out that the pathogenic mechanisms linking depression to diabetes require deeper exploration, a higher risk of diabetes was identified in depressed versus non-depressed subjects. The use of antidepressant drugs and untreated depression were also identified as risk factors for diabetes.

The DAWN (Diabetes Attitudes Wishes and Needs) study found that diabetes behaves as the cause of multiplex psychosocial problems, which in turn creates obstacles to achieving adequate glycemic control, as these interfere with self-care behaviors such as adherence to treatment.^7^ Pouwer et al.^8^ found that people with DM who are depressed are more likely to exercise poor glycemic control. Moreover, non-adherence to pharmacological treatment is also high among patients with type 2 DM (T2DM) and depression, in comparison with those free from symptoms of depression or any other alteration of their emotional state.^9^

It is very important to identify mental health problems in older people with T2DM, as these problems may interfere substantially with the general management of diabetes, as well as complications such as nephropathy or retinopathy.^10^ Adherence to pharmacological treatment thus becomes an essential aspect to be addressed when treating the population living with this disease, focusing especially on adults over 65 years, as this vulnerable group presents additional risk factors that hinder and in some cases preclude treatment, and lead to higher non-adherence.^11^ In our context, there are no studies that have jointly addressed these psychosocial problems in adherence to pharmacological treatment and control of T2DM in the elderly population treated in Primary Health Care.

For the above reasons, the aim of this study was to explore the influence of anxiety or depression symptoms as well as social risk in patients over 65 years of age with type 2 diabetes mellitus (T2DM) both in Non-adherence to pharmacological treatment and in the poor T2DM control.

## Material and methods

### Study design and participants

Cross-sectional descriptive study. The study population was made up of adults over 65 years of age with type 2 Diabetes Mellitus (T2DM) ascribed to the northern healthcare sector of Madrid, which comprises 35 primary care centers and 71 rural satellite centers. Of the population, 15.6% were over 65 years (11.6% of the men and 18.5% of the women),^12^ and their socioeconomic levels were heterogeneous, with a deprivation indexes of basic health zone between −1.79 and 0.95.^13^

### Data source and variables

The data for the study were obtained from the Madrid Primary Care Electronic Health Record (EHR) database in 2017. The records selected were those of patients over 65 years of age assigned to the centers belonging to the northern healthcare sector featuring a diagnosis of T2DM with the code T90 as per the International Primary Healthcare Classification version 2 in Spanish.^14^ The selection resulted in 26,703 records, from which were excluded all those with incomplete data for the principal variables (Non-adherence to pharmacological treatment; poor T2DM control; and symptoms of depression or anxiety).

Two dependent variables were analyzed, both indicators of incorrect T2DM control:

- Non-adherence to pharmacological treatment (Non-AdhT): obtained from the EHR by means of the Morisky-Green test,^15^ with dichotomous response categories.
- Poor T2DM control: defined based on maximum glycated hemoglobin levels (HbA1c) registered in the EHR during the year in which the study was run. According to the latest update of the International Diabetes Federation's Clinical Practice Recommendations for Managing Type 2 Diabetes in Primary Care, poor control was established as an HbA1c value greater than or equal to 7%.^16^

As for independent variables, we included clinical variables, socio-demographic features and health related behaviors:

- Clinical variables: Symptoms of depression or anxiety (yes/no) were obtained using standardized questions from the Madrid Classification of Clinical Symptoms that are included in the EHR. These questions were asked by a health professional in consultation. A “yes” was considered if there was a single positive response for both depression and anxiety^17^; time elapsed since T2DM was diagnosed, as a continuous and categorical variable (≤5 years, 6–10 years, 11–15 years, >5 years); body mass index, as a continuous and categorical variable according to the WHO (low weight or normal weight, overweight, obesity).^18^
- Sociodemographic variables: sex; age, as a continuous and categorical variable (66–79 years, 80–84 years, 85 years or over); region of origin (Spain or OECD countries, other countries); background of social risk (no, yes), measured through the indicators contemplated in the Madrid Primary Care EHR (living alone or with family members with limited capacity for providing support, in a conflictive family relationship, with family members who have difficulty taking patient care responsibilities, inadequate or deficient personal hygiene conditions, in living quarters that are inadequate for the patient's needs and lack of economic resources; a single positive indicator in the last two years is considered as a social risk); socioeconomic situation in the area of residence, measured through the deprivation index of basic health zone for the year 2011 to which the center belongs (categorized in quartiles, from better to worse socioeconomic situation).^13^
- Behaviors: practices some physical exercise, whether through sport, physical exercise, walking or strolling (no, yes).

Non-adherence to treatment was also used as a dependent variable in the analysis of factors associated to poor T2DM control.

### Statistical analysis

Patients’ characteristics were described using measures of central tendency (mean and standard deviation –SD–) and distribution of frequencies, following which the prevalence of treatment non-adherence and poor T2DM control were calculated. Bivariate logistic regression models were adjusted to conduct an analysis of the relationships between each of the clinical and sociodemographic characteristics and control of the disease. Multivariate logistic regression models were performed to include from the outset the variables with a *p* value of <0.2 in the bivariate analysis,^19^ as well as age and deprivation index quartile as adjustment variables. In order to incorporate a gender perspective, all the analyses were conducted globally and separately for men and women. Interaction terms were entered in the global models (sex and symptoms in the non-AdhT model; sex and non-AdhT in the poor T2DM control model). Prevalence and odds ratios were presented with confidence intervals of 95%.

### Ethical considerations

For the study, approval was obtained from the Universidad Autónoma de Madrid Ethics Committee (CEI 77 – 1415), and from the Central Research Commission of the Madrid Primary Care Service (96/16). For access to the Electronic Health Record, the data protection regulations were complied with: Regulation (EU) 2016/679 of the European Parliament and of the Council of 27 April 2016 on the protection of natural persons with regard to the processing of personal data and on the free movement of such data; and Organic Law 3/2018 of December 5, Protection of Personal Data and guarantee of digital rights.

**Flowchart of study population**. 

## Results

As can be seen in the flowchart of the study population, 26,703 subjects over 65 years of age had a diagnosis of T2DM in EHR, but the registration of the main variables (HBA1c values, Morisky–Green test, symptoms depression or anxiety and social risk) was complete in 884 patients. Of these, 64.7% (*n* = 572) were women. The mean age was 83.9 (SD: 5.5); 38.8% (*n* = 121) of the men and 49.7% of the women (*n* = 284) (*p* = 0.005) were 85 or older. 98.8% (*n* = 873) were autochthonous. A background of social risk was more common in women (35.7%, *n* = 201) than in men (19.0% *n* = 59) (*p* < 0.001). As for sociodemographic status in the area of residence (deprivation index), 36.7% (*n* = 210) of the women and 40.4% (*n* = 126) of the men (*p* = 0.028) were in the quartile 2. With regard to body mass index, the mean was 28.9 (SD: 4.8), obesity was found in 41.4% (*n* = 166) of the women and 30.7% (*n* = 79) of the men (*p* = 0.010). Physical activity was engaged in by 84.1% (*n* = 232) of the men and 65.6% (*n* = 307) of the women (*p* < 0.001). The mean time with diabetes was 12.3 years (SD: 7.8); those living with this disease for the last 11 to 15 years were 38.6% (*n* = 341). Symptoms of anxiety or depression were observed in 29% (*n* = 166) of the women and 18% (*n* = 56) of the men (*p* < 0.001) (Table 1).

**Table 1:** Patient characteristics, global and by sex.

	Global	Men	Women
*Sex*	884		
Men	35.3 (312)		
Women	64.7 (572)		

*Age group*	884		*p* = 0.005
66–79	23.4 (207)	28.2 (88)	20.8 (119)
80–84	30.8 (272)	33.0 (103)	29.6 (169)
85+	45.8 (405)	38.8 (121)	49.7 (284)

*Region of origin*	884		*p* = 0.478
Spain + OECD	98.8 (873)	98.4 (307)	99.0 (566)
Other countries	1.2 (11)	1.6 (5)	1.1 (6)

*Background of social risk*	874		*p* < 0.001
No	70.2 (614)	81.0 (252)	64.3 (362)
Yes	29.8 (260)	19.0 (59)	35.7 (201)

*Deprivation index quartile*	884		*p* = 0.028
Group 1	24.2 (214)	27.9 (87)	22.2 (127)
Group 2	38.0 (336)	40.4 (126)	36.7 (210)
Group 3	25.9 (229)	23.1 (72)	27.5 (157)
Group 4	11.9 (105)	8.6 (27)	13.6 (78)

*Body mass index*	658		*p* = 0.010
Low weight/normal weight	21.9 (144)	21.8 (56)	21.9 (88)
Overweight	40.9 (269)	47.5 (122)	36.7 (147)
Obesity	37.2 (245)	30.7 (79)	41.4 (166)

*Physical activity*	744		*p* < 0.001
No	27.6 (205)	15.9 (44)	34.4 (161)
Yes	72.4 (539)	84.1 (232)	65.6 (307)

*Time with diabetes*	884		*p* = 0.396
≤5 years	15.7 (139)	16.0 (50)	15.6 (89)
6–10 years	23.0 (203)	24.7 (77)	22.0 (126)
11–15 years	38.6 (341)	39.7 (124)	37.9 (217)
>15 years	22.7 (201)	19.6 (61)	24.5 (140)

*Depression/anxiety symptoms*	884		*p* < 0.001
No	74.9 (662)	82.0 (256)	71.0 (406)
Yes	25.1 (222)	18.0 (56)	29.0 (166)

**Figure 1:**
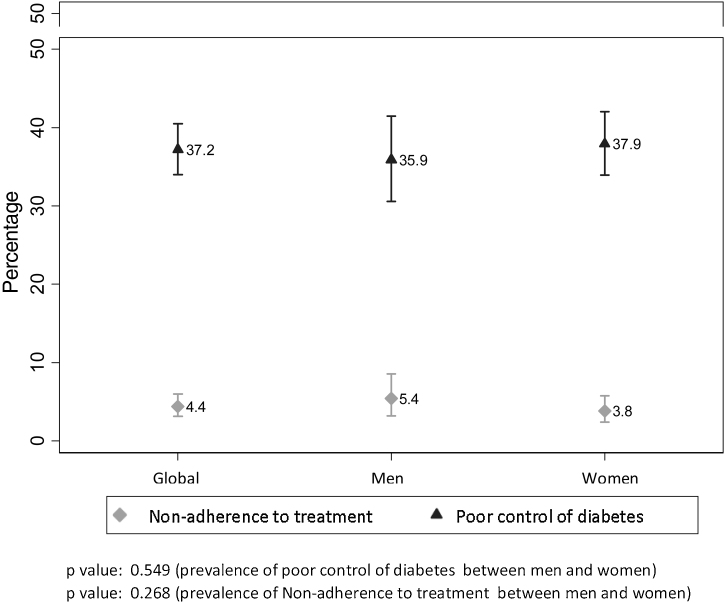
Prevalence of non-adherence to pharmacological treatment and poor Type 2 Diabetes Mellitus control, with 95% confidence interval, global and by sex.

The prevalence of Non-AdhT was 4.4% (*n* = 39; [95%CI: 3.2–5.9]); 5.4% (*n* = 17; [95%CI: 3.2–8.6]) in men and 3.8% (*n* = 22; [95%CI:2.4–5.8]) in women. Regarding the prevalence of poor T2DM control, this was identified in 37.2% (*n* = 329; [95%CI: 34–40.5]); 35.9% (*n* = 217; [95%CI: 30.57–41.49]) men and 37.9% (*n* = 217; [95%CI: 33.9–42]) women (Fig. 1).

### Non-adherence to pharmacological treatment

In the bivariate analysis, taking a *p* value of <0.2 for statistical significance, being at social risk raised the likelihood of Non-AdhT both globally (OR: 2.10; 95%CI: 1.10–4.01), and in men (OR: 2.48; 95%CI: 0.88–7) and women ((OR: 2.23; 95%CI: 0.95–5.27); the same was found for not performing physical exercise (global OR: 2.96; 95%CI: 1.43–6.1); men OR: 4.42; 95%CI: 1.45–13.45; women OR: 2.84; 95%CI: 1.06–7.60); and having symptoms of anxiety or depression (global OR: 2.41; 95%CI: 1.26–4.63; men OR: 4.57; 95%CI: 1.68–12.45) (Table 2).

**Table 2:** OR for non-adherence to pharmacological treatment based on the characteristics of the sample for each sex and global.

	Men			Women			Global		
	OR	95%CI	*P* value	OR	95%CI	*P* value	OR	95%CI	*P* value
*Sex (men)*									
Women							1.44	0.75–2.76	0.270

*Age group (85 and more)*									
66–79	1.15	0.34–3.91	0.817	1.91	0.69–5.25	0.210	1.60	0.73–3.48	0.237
80–84	1.19	0.37–3.79	0.774	1.12	0.39–3.22	0.826	1.20	0.55–2.61	0.645

*Social risk (No)*									
Yes	2.48	0.88–7.00	0.086	2.23	0.95–5.27	0.066	2.10	1.10–4.01	0.025

*Deprivation Index Quartile (Group 1)*									
Group 2	1.04	0.28–3.79	0.956	1.35	0.46–3.98	0.587	1.21	0.53–2.77	0.646
Group 3	2.23	0.63–7.96	0.215	0.97	0.29–3.25	0.960	1.37	0.57–3.28	0.478

*Body mass index (overweight)*									
Low weight/normal weight	1.09	0.19–6.15	0.920	1.71	0.48–6.08	0.407	1.48	0.54–4.05	0.450
Obesity	2.42	0.66–8.88	0.181	1.44	0.46–4.50	0.532	1.75	0.74–4.12	0.200

*Physical activity (yes)*									
No	4.42	1.45–13.45	0.009	2.84	1.06–7.60	0.038	2.96	1.43–6.10	0.003

*Time with diabetes (≤10 years)*									
11–15 years	0.63	0.20–1.97	0.421	1.33	0.45–3.91	0.600	0.93	0.43–2.01	0.850
>15 years	1.04	0.30–3.61	0.946	2.11	0.72–6.22	0.175	1.49	0.67–3.28	0.325

*Depression/anxiety symptoms (No)*									
Yes	4.57	1.68–12.45	0.003	1.73	0.73–4.14	0.215	2.41	1.26–4.63	0.008

The reference category of each variable is shown in parentheses; 95% CI: 95% confidence interval.

In the multivariate logistic regression models, adjusted for age and deprivation index in the area of residence, men who did not practice any physical exercise were 3.45 times more likely to present Non-AdhT [95%CI: 1.04–11.46], and having symptoms of anxiety or depression multiplied this by 3.88 [95%CI: 1.15–13.07]. In women, Non-AdhT was increased by social risk (OR: 5.61; 95%CI: 1.86–16.94) and not practicing physical exercise (OR: 4.07; 95%CI: 1.43–11.59). The global model also showed the highest risk of presenting Non-AdhT in men with symptoms of anxiety or depression (OR: 4.07; 95%CI: 1.32–12.59) (Table 3).

**Table 3:** Multivariate logistic regression models of non-adherence to pharmacological treatment.

Men (*n* = 276)	OR	95%CI	*P* value
*Age group (85 and more)*			
66–79	2.41	0.56–10.30	0.236
80–84	1.79	0.43–7.52	0.424

*Deprivation Index Quartile (Group 1)*			
Group 2	0.74	0.17–3.25	0.695
Groups 3–4	1.70	0.43–6.67	0.447

*Physical activity (yes)*			
No	3.45	1.04–11.46	0.044

*Depression/anxiety symptoms (no)*			
Yes	3.88	1.15–13.07	0.029

Women (*n* = 463)	OR	95%CI	*P* value
*Age group (85 and more)*			
66–79	2.21	0.66–7.41	0.200
80–84	1.12	0.32–3.88	0.863

*Deprivation Index Quartile (Group 1)*			
Group 2	0.89	0.27–2.95	0.848
Group 3	0.34	0.08–1.39	0.135

*Social risk (no)*			
Yes	5.61	1.86–16.94	0.002

*Physical activity (yes)*			
No	4.07	1.43–11.59	0.009

Global (*n* = 738)	OR	95%CI	*P* value
*Age group (85 and more)*			
66–79	2.22	0.88–5.59	0.090
80–84	1.39	0.55–3.52	0.486

*Deprivation Index Quartile (Group 1)*			
Group 2	0.81	0.32–2.05	0.662
Group 3	0.73	0.28–1.90	0.513

*Social risk (No)*			
Yes	3.01	1.38–6.54	0.005

*Physical activity (Yes)*			
No	3.02	1.38–6.64	0.006

*Sex and symptoms (women without symptoms)*			
Men without symptoms	1.52	0.57–4.08	0.401
Men with symptoms	4.07	1.32–12.59	0.015
Women with symptoms	1.18	0.42–3.33	0.759

The reference category of each variable is shown in parentheses; 95% CI: 95% confidence interval.

### Poor T2DM control

For the bivariate analysis, considering a *p* value of <0.2 for statistical significance, a higher likelihood of poor T2DM control was observed in overweight or obese patients as compared with normal or underweight patients, both globally (overweight OR: 1.59 [95%CI: 1.02–2.48]; obesity OR: 1.71 [95%CI: 1.09–2.67]) and in men (overweight OR: 1.86 [95%CI: 0.88–3.89]; obesity OR: 2.63 [C95%: 1.21–5.73]) and women (overweight OR: 1.51 [95%CI: 0.86–2.65]; in the absence of physical activity among men (OR: 1.97; 95%CI: 1.03–3.78); in patients having lived for a longer period with diabetes (>15 years as opposed to less than 10 years: globally (OR: 2.60 [95%CI:1.8–3.77]; men (OR: 2.69 [95%CI:1.41–5.12]; women (OR: 2.57 [95%CI: 1.63–4.06]); and in the absence of AdhT (global OR: 1.64 [95%CI: 0.86–3.12]; men OR: 4.68 [95%CI: 1.60–13.65]) (Table 4).

**Table 4:** OR for poor Type 2 Diabetes Mellitus control based on the characteristics of the sample for each sex and global.

	Men			Women			Global		
	OR	95%CI	*P* value	OR	95%CI	*P* value	OR	95%CI	*P* value
*Sex (men)*									
Women							1.09	0.82–1.45	0.549

*Age group (66–79)*									
80–84	1.31	0.72–2.38	0.383	1.01	0.62–1.65	0.958	1.13	0.77–1.64	0.540
85 y más	1.27	0.71–2.27	0.422	1.08	0.69–1.68	0.740	1.16	0.82–1.65	0.398

*Social risk (no)*									
Yes	1.07	0.59–1.93	0.821	0.77	0.54–1.11	0.163	0.86	0.63–1.16	0.328

*Deprivation Index Quartile (Group 4)*									
Group 1	1.06	0.60–1.87	0.853	0.91	0.58–1.42	0.666	1.24	0.76–2.03	0.386
Group 2	1.28	0.67–2.44	0.452	0.82	0.51–1.33	0.424	1.20	0.76–1.90	0.439
Group 3	0.80	0.31–2.04	0.641	0.76	0.43–1.37	0.366	1.20	0.74–1.96	0.456

*Body mass index (low weight/normal weight)*									
Overweight	1.86	0.88–3.89	0.102	1.51	0.86–2.65	0.146	1.59	1.02–2.48	0.039
Obesity	2.63	1.21–5.73	0.015	1.35	0.78–2.34	0.290	1.71	1.09–2.67	0.020

*Physical activity (yes)*									
No	1.97	1.03–3.78	0.040	0.72	0.48–1.08	0.114	0.95	0.68–1.33	0.776

*Time with diabetes (*≤*10 years)*									
11–15 years	2.07	1.21–3.55	0.008	2.61	1.74–3.93	0.000	2.40	1.74–3.33	0.000
>15 years	2.69	1.41–5.12	0.003	2.57	1.63–4.06	0.000	2.60	1.80–3.77	0.000

*Depression/anxiety symptoms (no)*									
Yes	0.99	0.54–1.81	0.975	0.83	0.57–1.22	0.345	0.89	0.65–1.22	0.458

*Non-adherence to pharmacological treatment (no)*									
Yes	4.68	1.60–13.65	0.005	0.76	0.30–1.88	0.548	1.64	0.86–3.12	0.132

The reference category of each variable is shown in parentheses; 95% CI: 95 confidence intervals.

In the multivariate logistic regression models, adjusted for age and deprivation index in the area of residence, poor T2DM control was increased among men by being overweight (OR: 2.09; 95%CI: 0.96–4.53) or obese (OR: 3.81; 95%CI: 1.61–9.02); the length of time living with T2DM (from 11 to 15 years OR: 2.69 [95%CI: 1.40–5.17]; more than 15 years OR: 3.43 [95%CI: 1.60–7.37]; Ref: less than10 years); and Non-AdhT (OR: 3.53; 95%CI: 1.04–12.02). In women, only the time spent living with T2DM increased the risk of poor control (from 11 to 15 years OR: 2.67 [95%CI: 1.77–4.05]; more than 15 years OR: 2.64 [95%CI: 1.67–4.18]; Ref: less than 10 years). The global model also showed a statistically significant risk of poor T2DM control in men with Non-AdhT (OR: 3.75; 95%CI: 1.12–12.60) (Table 5).

**Table 5:** Multivariate logistic regression models of poor control of Type 2 Diabetes Mellitus.

Men (*n* = 257)	OR	95%CI	*P* value
*Age group (66–79)*			
80–84	1.55	0.77–3.13	0.223
85 and more	1.42	0.69–2.92	0.341

*Deprivation Index Quartile (Group 4)*			
Group 1	0.84	0.30–2.37	0.736
Group 2	0.81	0.30–2.21	0.682
Group 3	0.55	0.18–1.65	0.289

*Body mass index (low weight/normal weight)*			
Overweight	2.09	0.96–4.53	0.063
Obesity	3.81	1.61–9.02	0.002

*Time of diabetes (≤10 years)*			
11–15 years	2.69	1.40–5.17	0.003
>15 years	3.43	1.60–7.37	0.002

*Non-adherence to pharmacological treatment*	3.53	1.04–12.02	0.043
Women (*n* = 468)	OR	95%CI	*P* value
*Age group (66–79)*			
80–84	0.92	0.56–1.51	0.801
85 and more	1.1	0.68–1.68	0.686
*Deprivation Index Quartile (Group 4)*			
Group 1	1.38	0.76–2.52	0.495
Group 2	1.28	0.73–2.23	0.602
Group 3	1.09	0.61–1.95	0.627
*Time with diabetes (≤10 years)*			
11–15 years	2.67	1.77–4.05	0.000
>15 years	2.64	1.67–4.18	0.000
Global (*n* = 658)	OR	95%CI	*P* value
*Age group (66–79)*			
80–84	0.98	0.63–1.51	0.91
85 and more	1.15	0.75–1.75	0.527
*Deprivation Index Quartile (Group 4)*			
Group 1	0.97	0.53–1.77	0.924
Group 2	0.92	0.52–1.63	0.781
Group 3	0.58	0.32–1.07	0.082
*Body mass index (low weight/normal weight)*			
Overweight	1.74	1.10–2.75	0.018
Obesity	2.12	1.32–3.41	0.002
*Time with diabetes (≤10 years)*			
11–15 years	2.71	1.83–4.02	0.000
>15 years	2.88	1.83–4.54	0.000
*Sex and Non-adherence to pharmacological treatment (men with adherence)*			
Men with non-adherence	3.75	1.12–12.60	0.033
Women with adherence	1.19	0.83–1.70	0.333
Women with non-adherence	0.88	0.31–2.50	0.804

The reference category of each variable is shown in parentheses; 95% CI: 95% confidence interval.

## Discussion

Our results show that symptoms of depression or anxiety (in men) were associated with No-AdhT and that this, in turn, was associated with poor control of T2DM. This sheds light on the fact that treatment adherence may play a role as mediator between emotional condition and control over chronic problems. Xue et al.^20^ demonstrated the intermediary role of treatment adherence in controlling arterial hypertension in older adults suffering depression. On the one hand, the relationship found in this study between experiencing symptoms of anxiety or depression and higher percentages of non-adherence to medicament treatment is consistent with other studies highlighting the link between mental health problems, such as depression, and non-compliance with medicament therapy, in people with diabetes,^9^, ^11^, ^21^ and with other health problems such as infection with HIV.^22^ On the other hand, there is also a solid relationship between treatment adherence and better control over diabetes.^23^

No direct association was found between symptoms of anxiety or depression and control of DM2, as other studies have shown.^7^, ^8^, ^24^ This could be related to the use of secondary data that did not allow the incorporation of more robust variables such as the diagnosis of anxiety or depression. Despite this, we thought it relevant, and innovative in our context, to maintain symptoms of anxiety or depression on the basis that their presence accounts for clinically relevant psycho-emotional problems, especially in community settings where depression and anxiety remain largely undiagnosed, such as it has been pointed out in other studies that the symptoms of these diseases have also been used.^25^

Our results reflect that in women, social risk has a negative impact on treatment adherence, which is in line with the findings of other studies pointing out that social isolation, that is, the lack of support networks such as family and friends, is a risk factor that accumulates throughout a person's life and emerges as a negative influence especially in chronic older adult patients, raising the likelihood of poor self-care particularly where pharmacological therapy is concerned.^26^, ^27^ Although there is only scarce research linking social risk to non-adherence to medication treatment, Oates et al.^28^ shows that people exposed to three or more social risk factors (low income and chronic stress, in particular), are up to three times more likely to fail to adhere to their treatment than those free from social risk factors. Kususlan et al.^29^ demonstrated that higher scores on a loneliness scale were associated with greater difficulties in adhering to medication therapy, while it is important to underscore that loneliness is a social risk indicator, especially among the older people.

This work reveals differences between men and women that may constitute gender inequality. The association between symptoms of anxiety or depression and non-adherence to treatment, and between the latter and poor T2DM control, appeared only in the case of men, whereas social risk and treatment non-adherence were only associated in women. The traditional masculine attitude is influential in seeking help and applying risk management in men who feel the onset of disease,^30^ while there is evidence that men take fewer preventive measures or medical check-ups.^31^ Dale et al.^32^ found that men with diabetes and coronary disease showed poorer compliance with their cardiac rehabilitation program, despite a poorer prognosis, than women. Moreover, the review by Emslie^33^ reflected that wives encourage their husbands to seek medical help when symptoms appear, but do not communicate their own symptoms to their husbands to prevent them from worrying. In this analysis, we can also highlight, from a gender perspective, the higher prevalence of social risk indicators among women, such as the availability of technical or personal assistance for basic everyday tasks, which is lower in women over 65 years than in men in the same age group despite their needs being greater, most especially in lower-income homes.^34^

Among the main limitations of this study are those derived from the use of documentary sources, which has caused the loss of subjects due to the incompleteness of the EHR for the main variables, as well as the non-inclusion of other potentially confounding variables, such as comorbidities or medication and its tolerance, given its low registration. It could be that it had also caused less precision in the detection of symptoms of anxiety or depression, but it should be noted as a strength, that it was a health professional who detected them during the consultation. On the other hand, it should be highlighted that the implementation of a single EHR on a centralized database has allowed the regulation and standardization of recording systems; it should be mentioned that the Madrid Primary Care EHR has proven to be a valid and reliable source of information for the epidemiological surveillance of diabetes mellitus.^35^

It is also important to note that, although current recommendations on glycemic control in older people have raised HbA1c levels between 7.5 and 8%, in the present study values lower than 7% were used to establish good control, which could contribute to an overestimation of poor control. We made this decision taking into account the criteria of the International Diabetes Federation, which only recommend higher levels (7.5–8%) in the special population: “in patients using multiple medications including glucose lowering drugs where predicted survival is short (e.g., <10 years), with cognitive impairment, chronic kidney disease, or severe cardiovascular disease associated with multiple comorbidities; patients with these conditions should be referred to specialized care”.^16^ In many subjects in our study, they either did not meet these characteristics or it was not possible to know, they were also treated in Primary Health Care.

The fact that only 28% of the subjects had undergone the Morisky-Green test could lead to a detection bias. In this regard, it should be mentioned that the starting population, the 26,703 people with T2DM over 65 years of age, were slightly younger (mean age 77.4 years, compared to 83.9 in the studied population), with less proportion of women (51% versus 64.7%) and with a higher proportion in quartile 4 regarding the deprivation index (22.5% versus 11.5%), but in other variables such as the body mass index, the practice of exercise or the time with diabetes were quite similar. Thus, in terms of the external validity of the study, it is necessary to take into account these considerations, and that the findings are especially applicable to large elderly treated in Primary Health Care.

Another limitation of this study is the cross-sectional nature of its design, which does not allow us to guarantee the premise of temporal precedence to establish a causal relationship; and that on the other hand, it could also lead to an overestimation of the effect, since the presence of an HbA1c ≥7% and the presence of these psychosocial factors could be treated as isolated events.

More research is needed to delve into the gender differences found in this work, and make it possible to identify, through longitudinal designs and using primary data, more solid causal relationships, as well as confirm the role of adherence to treatment as a mediator between psychosocial problems and control of diabetes mellitus. In addition, with larger sample sizes, it would be interesting to perform a stratified analysis by age groups applying different cut-off points for HbA1c.

In conclusion, although our findings do not allow us to prove that symptoms of anxiety or depression are associated with poor T2DM control in patients over 65 years of age, they do show that these symptoms are observed as a risk factor to Non-AdhT in men, while social risk has the same effect in women. Moreover, Non-AdhT increases the risk of poor T2DM control, which may be related to an intermediary role between psychosocial factors and poor T2DM control. From a gender perspective, it is important to detect psychosocial problems in older adults with diabetes and to reinforce strategies to improve their adherence to drug treatment in these patients.

**What is known about the subject**

• Mental health problems in elderly patients with type 2 diabetes mellitus (T2DM) can substantially interfere with the control of T2DM.
• Furthermore, non-adherence to drug treatment (Non-AdhT) is high among patients with T2DM and depression or other alterations in their emotional state.
• It is relevant to identify psychosocial problems in older people with T2DM, since these problems can substantially interfere with the management of diabetes and its complications.

**What this study contributes**

• In people older than 65 years with T2DM, although Non-AdhT is low, the prevalence of poor T2DM control is high.
• Symptoms of depression or anxiety are a risk factor to Non-AdhT in men, while social risk has the same effect in old women.
• Non-AdhT in old men increases the risk of poor T2DM control, which may be related to an intermediary role between mental health problems and poor T2DM control.

## Ethical considerations

For the study, approval was obtained from the Universidad Autónoma de Madrid Ethics Committee (CEI 77 – 1415), and from the Central Research Commission of the Madrid Primary Care Service (96/16). For access to the Electronic Health Record, the data protection regulations were complied with: Regulation (EU) 2016/679 of the European Parliament and of the Council of 27 April 2016 on the protection of natural persons with regard to the processing of personal data and on the free movement of such data; and Organic Law 3/2018 of December 5, Protection of Personal Data and guarantee of digital rights.

## Funding statement

The present work has not received any funding.

## Conflict of interest

The authors have no conflict of interest to declare.
